# The Doctor as a Popular Leader

**Published:** 1892-11

**Authors:** 


### The Doctor as a Popular Leader.

A statement having been made in a German professional work to the effect that Mr. Gladstone had prophesied that doctors would become the leaders of the people, a correspondent wrote to the Prime Minister for the purpose of ascertaining the truth of the matter. In reply Mr. Gladstone said: "So far as regards the exact words cited in your letter, I cannot positively say aye or no, and I rather think that I should in using them have added some qualifying or limited expressions. But it is certainly a fact that for a very long time I have believed the medical profession to be both in a state of absolute advance from the progress of its science—this, it may be said, is mere commonplace—and of relative advance from the particular features attaching to our civilization in its onward movement."—*N. Y. Med. Record.*

Effects of variously colored lights on insane persons have been observed by the director of the Milan (Italy) Insane Asylum. A melancholy patient in a rosy light improved perceptibly in twelve hours. In twenty-four hours he called for food, although for many preceding days nourishment had been administered to him against his will. Thereupon the director had rooms furnished in solid colors and confined patients to them. Green and blue were found to be the most quieting, rose the most cheering, red the most exciting. All the patients in the asylum will be confined hereafter in apartments furnished in colors to suit the nature of their maladies.

